# The incidence and prevalence of juvenile idiopathic arthritis differs between ethnic groups in England

**DOI:** 10.1093/rheumatology/kead700

**Published:** 2023-12-22

**Authors:** Richard P Beesley, Kimme L Hyrich, Jenny H Humphreys

**Affiliations:** Centre for Epidemiology Versus Arthritis, The University of Manchester, Manchester Academic Health Science Centre, Manchester, UK; Centre for Epidemiology Versus Arthritis, The University of Manchester, Manchester Academic Health Science Centre, Manchester, UK; National Institute of Health Research, Manchester Biomedical Research Centre, Manchester University NHS Foundation Trust, Manchester, UK; Centre for Epidemiology Versus Arthritis, The University of Manchester, Manchester Academic Health Science Centre, Manchester, UK; National Institute of Health Research, Manchester Biomedical Research Centre, Manchester University NHS Foundation Trust, Manchester, UK

**Keywords:** ethnicity, equity, juvenile idiopathic arthritis, incidence, clinical practice research datalink

## Abstract

**Objectives:**

JIA is a heterogeneous group of rare autoimmune disorders characterized by chronic joint inflammation of unknown aetiology with onset under 16 years. Accurate estimates of disease rates help understand impacts on individuals and society, and provide evidence for health service planning and delivery. This study aimed to produce the first national estimates of incidence and prevalence by ethnic group using electronic health records.

**Methods:**

Data from the Clinical Practice Research Datalink Aurum, a primary care electronic health record database in England, were used to estimate the incidence and prevalence of JIA by ethnic group amongst children and young people aged under 16 years between 2003 and 2018, with cases validated using Hospital Episode Statistics. χ^2^ was used to test the difference in proportions compared with the ethnic distribution of England.

**Results:**

A total of 424 incident cases of JIA were identified, 389 validated using Hospital Episode Statistics records. Incidence of JIA was higher amongst those of white ethnic group (6.2 per 100 000 population) compared with mixed (3.0 per 100 000), Asian (2.7 per 100 000) and Black (2.9 per 100 000) communities. The ethnic group distribution of cases differed significantly compared with the general population (*P* < 0.0001).

**Conclusion:**

The incidence and prevalence of JIA differs between ethnic groups, and is different from the general population. This is likely to be due to a combination of genetic and equity factors. Further research to understand the underlying cause of these differences is important to enable targeted interventions and appropriate service provision.

## Graphical abstract

**Figure:**
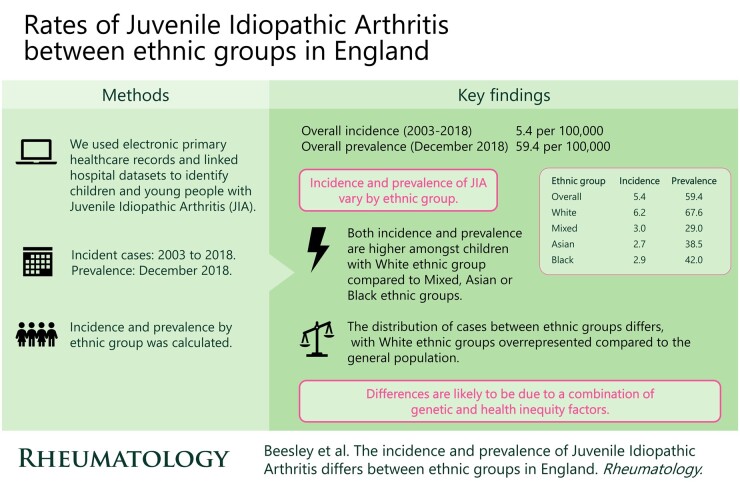

Rheumatology key messages

- Incidence and prevalence of JIA differs between ethnic groups.
- Rates of JIA in the UK are highest amongst people from white ethnic groups.
- Understanding drivers of differences in disease occurrence can enable targeted interventions and service provision.

## Introduction

JIA is a heterogeneous group of autoimmune disorders characterized by chronic joint inflammation of unknown aetiology with onset <16 years [1]. JIA can be associated with joint erosion, chronic pain and lifelong disability, as well as extra-articular comorbidities such as uveitis; this has implications for long-term physical and mental health as well as employment prospects and family life [2, 3].

Estimates of the incidence and prevalence of JIA vary between countries and differ by study methods [4]. A study in 2021 estimated the annual incidence and prevalence of JIA in the UK [5] as 5.6 and 43.5 per 100 000 population, respectively. Accurate estimates of disease rates are important in order to understand disease impacts on individuals and society, as well as providing evidence for healthcare service planning and delivery.

Whilst some studies have suggested differences in incidence and/or prevalence between ethnic groups in New Zealand [6], Slovakia [7] and Canada [8], these have been derived from hospital-derived cohorts, meaning that there could be a bias in the representativeness of the patients included where some ethnic groups are less likely to attend hospital-based services. There have been no national estimates of incidence or prevalence by ethnic group using more complete health data, such as administrative health records. In the UK, almost all residents are registered with a primary care practitioner [General Practitioner (GP), or family doctor]. As such, electronic health records from primary care practitioners provide estimates of disease rates close to population levels.

There are two main reasons why differential rates of disease occurrence between ethnic groups may be identified. First, biological or genetic differences may have an impact; these have been associated in other autoimmune conditions such as RA which has higher rates amongst First Nations people in North America [9] and SLE which has higher rates amongst people of Black or Asian ethnicity [10]. Secondly, health inequities (avoidable systemic differences in health outcomes for different population groups [11]) may lead to specific ethnic groups being less likely to be referred resulting in delays in diagnosis and apparent differences in incidence and prevalence rates.

This study aimed to estimate the incidence and prevalence rates of JIA by ethnic group in primary care electronic health records in England. Ethnicity is associated with levels of deprivation [12], so indicators of deprivation and of population density (using a national urban/rural classification) were also considered.

## Methods

### Setting

The study was set in the Clinical Practice Research Datalink (CPRD) Aurum database in England. CPRD Aurum is a large national database of electronic primary care records, which obtains routinely collected data from primary care practices who participate. It covers ∼20% of the English population and is considered to be broadly representative in terms of age, gender and ethnicity [13]. Linkage to Hospital Episode Statistics (HES) data is also available for those practices who have consented to linkage. The HES dataset contains details of all inpatient admissions, including diagnoses, and outpatient clinics attended at English hospitals. Inpatient admission diagnoses are coded to the International Classification of Diseases (ICD). Outpatient clinic appointment records contain the date, appointment type and clinic speciality but not diagnoses. Given private practice and treatment solely in non-speciality clinics in paediatric rheumatology are rare in the UK, linkage to HES provides near-complete coverage. CPRD with HES linkage has been investigated for its ethnic representativeness and found to be broadly representative in terms of ethnic group and deprivation [13, 14]. The study period was from 1 January 2003 to 31 December 2018. The CPRD has pre-existing ethical approval from a National Research Ethics Committee, covering all observational research using anonymized CPRD data. The study was approved by the CPRD Independent Scientific Advisory Committee (ISAC) approval system (protocol number 19_060). All data in this study were anonymized by CPRD and therefore patient consents are exempted; select primary care general practices consent to submitting data to CPRD at a practice level with individual patients having the right to opt out.

### Study population

The study population was children and young people (CYP) <16 years of age registered with a GP practice in England in CPRD Aurum. Study entry point was the later of registration with a CPRD Aurum GP practice or 1 January 2003. Study end date was the earliest of leaving the GP practice, age 16 years, death or the end of the study period (31 December 2018). The CPRD holds only the year of birth, therefore all CYP were assigned 1 January as their date of birth.

### Case definition and validation

Cases of JIA were identified using two previously developed Read code lists [5]. Briefly, three rheumatologists (including one paediatric rheumatologist) reviewed and identified potential JIA codes in use in CPRD; CPRD does not separately code ILAR subtypes of JIA. The first code list included broad generic codes (such as ‘arthritis’) as well as specific codes (such as ‘juvenile arthritis’) and adult descriptions of diseases (such as ‘rheumatoid arthritis’). The second code list included only the specific codes for JIA.

Cases were validated using the linked HES dataset, available from 2003 for the subset of patients whose GP practices consented to the linkage. Cases from the broad code list were considered validated if (i) they had an inpatient admission (including day case admissions for joint injections or infusion therapies) for JIA according to ICD-10 codes (codes beginning M08 or M09), or (ii) had three or more outpatient appointments in either rheumatology and/or paediatric rheumatology clinics (Supplementary Figure S1).

Cases were recorded as incident if (i) they were aged under 16 years of age; (ii) they received a code before 31 December 2018; and (iii) either they were registered with a GP for at least 1 year prior to their first code, or they were aged under 3 years old when they received their first code. Cases aged >3 years but with less than 1 year registration were considered prevalent (Supplementary Figure S2).

### Ethnicity

Ethnic group is self-defined and captured and recorded by GP practices during registration and appointments, and in HES records during clinic attendances and hospital admissions. The HES dataset includes an overall field for ethnic group, based on all HES records for that patient—this contains the most frequently cited ethnic group for a patient, unless that category is ‘unknown’ in which case the second most frequently cited ethnic group is stored. There are many reasons why ethnic group may not be asked and/or stored during appointments. Each patient in CPRD may have their ethnic group collected and stored multiple times. Using the same methodology as HES, an overall ethnic group entry was calculated for each CYP.

Where linked HES records existed for a patient, their ethnicity as stored in HES was used for this study; in all other cases, or where HES records cited ‘unknown’ ethnic group, the overall CPRD record was used (fewer than 1% of cases). A comparison between HES and CPRD records, for those patients with linked records, showed few differences with >96% concordance. Ethnic group was aggregated using the Office for National Statistics (ONS) England ethnic group classification [15], which comprises 5 first-tier categories—white, mixed, Asian, Black and other. Given ‘other’ is a broad and heterogeneous group [16] they were excluded from detailed analysis.

### Other demographic information

In addition to age, gender and region stored within the CPRD, linked records provide an indicator of deprivation for each CYP using ONS Indices of Multiple Deprivation (IMD) quintiles assigned by home address postcode, along with a binary urban/rural classification at GP practice level. IMD is an indicator of relative deprivation based on small geographic areas, based on 37 different national datasets by the ONS.

### Statistical analysis

The characteristics of CYP who met the case definition were tabulated. Incidence rates overall and by ethnic group, indirectly standardized by age and region, were calculated as the number of new cases per 100 000 person-years. Prevalence rates overall and by ethnic group, indirectly standardized by age and region, were calculated using the number of CYP in the CPRD on 31 December 2018 as the denominator.

Estimates were indirectly standardized by age and region to ethnic groups using 2021 Census data to account for varying ethnic diversity across different age groups and regions of England.

Rates were calculated using specific and validated case definitions. The distribution of observed JIA cases across ethnic groups was subsequently compared with the expected distribution based on population statistics using χ^2^ analysis. Differences in IMD classification between ethnic groups was compared using χ^2^ analysis.

Given the upper limit for inclusion in the study was both age 16 years and December 2018, consideration was given as to whether the reason for difference between ethnic groups was a feature of the age of onset, with the latter being associated with ethnicity. As such, a sensitivity analysis of those aged 10–15 years was undertaken, separately from an analysis of younger patients.

Analysis was performed using Stata [17], with indirect standardization and χ^2^ analyses in Microsoft Excel [18].

## Results

### Overall

A total of 4 482 315 CYP were identified from CPRD Aurum. Of these, 48.1% were female.

### Cases

Between 1 January 2003 and 31 December 2018 there were a total of 424 incident cases amongst CYP under the age of 16 years using the specific code list definition, and 389 incident cases validated using HES records (Table 1). Cases were more frequently females (64.2% of specific cases, and 65.6% of validated cases). There was little difference between cases identified by the specific code list and validated cases in the distribution of any demographic measure considered. The majority of cases were from urban communities (88%), which is slightly higher than the England population (83% urban) [19]. Overall cases were broadly distributed equally across the five IMD quintiles.

**Table 1.** Baseline characteristics of incident cases, December 2018

Characteristics		Specific code list	Validated cases^a^
*n*		424	389
Gender, *n* (%)	Male	152 (35.8)	134 (34.4)
	Female	272 (64.2)	255 (65.6)
Region of England, *n* (%)	North East	34 (8.0)	33 (8.5)
	North West	74 (17.5)	68 (17.5)
	Yorkshire & The Humber	13 (3.1)	13 (3.3)
	East Midlands	13 (3.1)	12 (3.1)
	West Midlands	77 (18.2)	71 (18.3)
	East of England	19 (4.5)	19 (4.9)
	South West	63 (14.9)	62 (15.9)
	South Central	39 (9.2)	36 (9.3)
	London	66 (15.6)	55 (14.1)
	South East Coast	26 (6.1)	20 (5.1)
Urban/rural classification, *n* (%)	Urban	373 (88.0)	341 (87.7)
	Rural	51 (12.0)	48 (12.3)
IMD quintile, *n* (%)	1—least deprived	82 (19.3)	76 (19.5)
	2	89 (21.0)	78 (20.1)
	3	73 (17.2)	69 (17.7)
	4	90 (21.2)	82 (21.1)
	5—most deprived	89 (21.0)	83 (21.3)
Ethnic group	White	353 (83.3)	323 (83.0)
	Mixed	16 (3.8)	16 (4.1)
	Asian	27 (6.4)	26 (6.7)
	Black	14 (3.3)	13 (3.3)
	Other^b^	14 (3.3)	11 (2.8)

a Validated cases were identified from the broad code list.

b The ‘other’ ethnic group is a varied and heterogeneous group and so excluded from further analysis. IMD: Indices of Multiple Deprivation.

The proportion of CYP by ethnic group was not different between the specific code list definition and cases validated using HES records, with the majority of cases being white CYP (83%). Around 3% of incident cases by either definition were recorded as ‘other’ ethnic group; due to the heterogeneity of this group they were excluded from further detailed analysis.

### Incidence

**Figure 1.**
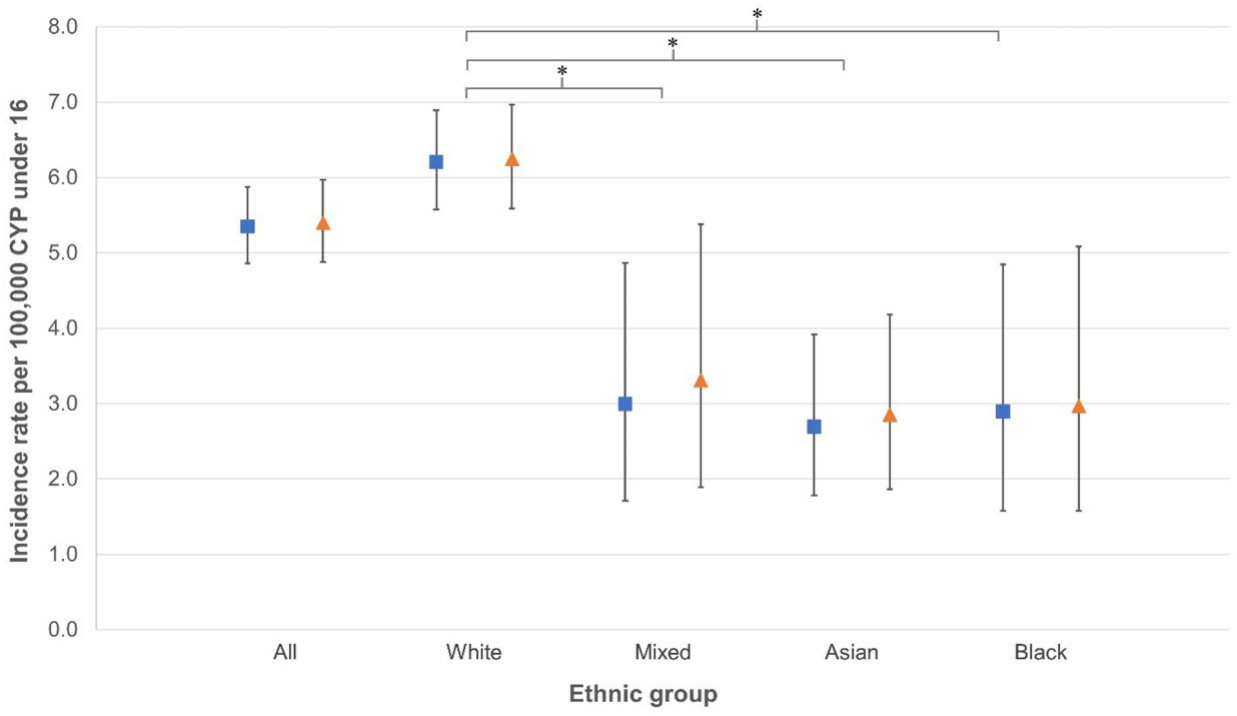
Incidence rate of JIA and 95% CIs, per 100 000 person years, of JIA-specific codes (blue squares) and HES-validated cases (orange triangles) of JIA amongst children and young people under the age of 16 years in December 2018, indirectly standardized by age and region, by ethnic group. ^*^Significantly lower incidence rate compared with ‘white’

The overall incidence rate was 5.4 per 100 000 person years (95% CI 4.9, 6.0) and varied by ethnic group (Table 2 and Fig. 1). The incidence rate for white CYP (validated cases 6.3 per 100 000 person years; 95% CI 5.6, 7.0) was higher than mixed CYP (3.3; 95% CI 1.9, 5.4), Black CYP (3.0; 95% CI 1.6, 5.1) and Asian CYP (2.9; 95% CI 1.9, 4.2).

**Table 2.** Incidence rates of JIA per 100 000 person-years amongst children and young people aged under 16 in December 2018 by ethnic group, indirectly standardized by age and region using ONS 2021 Census data

Incidence	All	White	Mixed	Asian	Black
JIA specific case, *n*	424	353	16	27	14
Incidence rate (95% CI)	5.4 (4.9, 5.9)	6.2 (5.6, 6.9)	3.0 (1.7, 4.9)^a^	2.7 (1.8, 3.9)^a^	2.9 (1.6, 4.9)^a^
Validated JIA cases, *n*	389	323	16	26	13
Incidence rate (95% CI)	5.4 (4.9, 6.0)	6.3 (5.6, 7.0)	3.3 (1.9, 5.4)^a^	2.9 (1.9, 4.2)^a^	3.0 (1.6, 5.1)^a^

Excludes children and young people whose ethnic group was recorded as ‘other’ due to the wide heterogeneity in that group.

a Significantly lower than ‘white’ ethnic group.ONS: Office for National Statistics.

The ethnic group distribution of cases differed significantly compared with the general population (*P* < 0.0001 χ^2^), with the observed: expected ratio ranging from 0.5:1 for CYP of mixed or Asian ethnic group to 1.1:1 for white CYP (Table 3).

**Table 3.** The proportion of incident JIA cases, defined by specific code list, amongst children and young people aged under 16 in December 2018 by ethnic group compared with the general population

Ethnic group	White	Mixed	Asian	Black
Proportion of CYP <16 years:				
England general 2021, %	72.4	7.0	12.3	5.6
JIA, %	83.3^a^	3.8^a^	6.4^a^	3.3^a^
95% CI	79.7, 86.8	2.0, 5.6	4.0, 8.7	1.6, 5.0
Observed: Expected ratio	1.1:1	0.5:1	0.5:1	0.6:1

a Statistically significant when compared with ethnic group distribution in general population, *P* <0.001 χ^2^. CYP: children and young people.

In the sensitivity analyses, the comparison of only those aged 10–15 years, and younger patients as a separate analysis (results not shown), the differences remain, indicating that the difference seen between ethnic groups is real and not a feature of age of onset.

### Prevalence

The overall prevalence rate in 2018 was 62.6 per 100 000 population (95% CI 58.2, 67.2) ≤16 years old and varied by ethnic group (Table 4). The prevalence rate for white CYP was higher (validated JIA cases 71.1 per 100 000 CYP; 95% CI 65.5, 77.0) than mixed CYP (29.4; 95% CI 19.1, 43.5), Asian CYP (42.1; 95% CI 32.4, 53.8) or Black CYP (46.3; 95% CI 31.8, 65.0).

**Table 4.** Prevalence of JIA amongst children and young people aged under 16 in December 2018 by ethnic group, indirectly standardized by age and region using ONS 2021 Census data

Prevalence	All	White	Mixed	Asian	Black
JIA-specific case, *n*	795	643	28	66	34
Prevalence rate per 100 000 (95% CI)	59.4 (55.3, 63.7)	67.6 (62.5, 73.0)	29.0 (19.3, 42.0)^a^	38.5 (29.7, 48.9)^a^	42.0 (29.1,58.7)^a^
Validated JIA cases, *n*	742	600	25	64	33
Prevalence rate per 100 000 (95% CI)	62.6 (58.2, 67.2)	71.1 (65.5, 77.0)	29.4 (19.1, 43.5)^a^	42.1 (32.4, 53.8)^a^	46.3 (31.8, 65.0)^a^

Excludes children and young people whose ethnic group was recorded as ‘other’ due to the wide heterogeneity in that group.

a Significantly lower than ‘white’ ethnic group.ONS: Office for National Statistics.

### Deprivation

**Figure 2.**
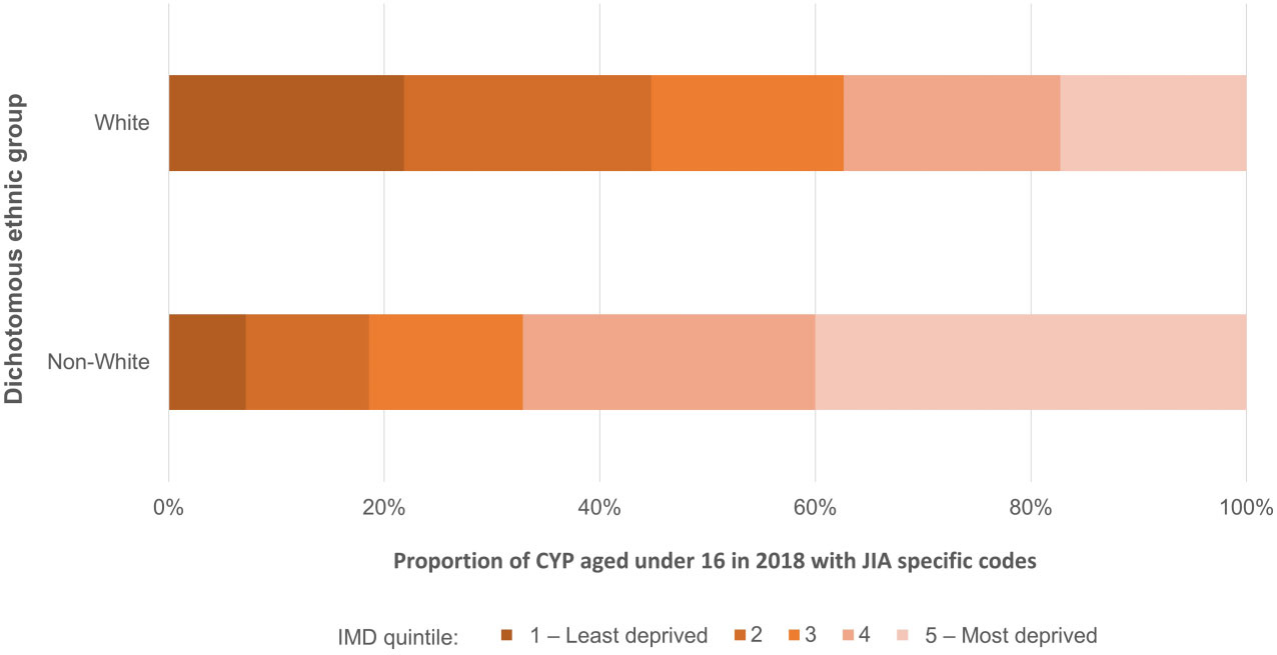
The proportion of incident cases of JIA specific codes amongst children and young people under the age of 16 in December 2018, by IMD quintile comparing dichotomous ethnic group classification

The proportion of incident cases by IMD quintile overall was broadly in line with the general population, with ∼20% of cases in each quintile. However, whilst the number of children and young people with JIA who have an ethnicity recorded as anything other than ‘white’ is small, the proportion by IMD quintile differed significantly between ethnic groups, with greater proportion of cases of JIA from non-white ethnicity coming from most deprived backgrounds. Comparing the cases from the 40% most deprived local areas with the least deprived 40% by dichotomous ethnic group classification (Fig. 2 and Table 5), there is a significantly higher proportion of cases amongst deprived communities from non-white ethnic groups (*P* = 0.0001, χ^2^).

**Table 5.** The distribution of incident JIA cases by dichotomous ethnic group for the most deprived 40% and least deprived 40% of local areas

	White*, n* (%)	Non-white, excl. other, *n* (%)
*N*	353	70
Least deprived 40%	158 (44.8)	13 (18.6)
Most deprived 40%	132 (37.4)	47 (67.1)

Distribution is statistically significantly different from expected values, *P* = 0.0001 χ^2^.

### Urban/rural indicator

There was no difference in the proportion of incident cases linked to GP practices in urban areas (88.0%) compared with the general population of CYP aged under 16 years in CPRD (88.5%).

## Discussion

This study has provided the first estimates of the incidence and prevalence of JIA by ethnic groups in England and the first estimates of the incidence and prevalence of JIA by ethnic group based on comprehensive national electronic health records. The 2018 overall age and region indirectly standardized incidence rate of JIA for CYP with white ethnicity was 6.2 per 100 000 person-years, significantly higher than for CYP of other ethnicities (range 2.7–3.0). Similarly, the 2018 overall age and region indirectly standardized prevalence rate of JIA for CYP with white ethnicity was 67.6 per 100 000 population, also higher than for CYP of other ethnicities (range 29.0–42.0).

A previous study reviewed medical records of children and young people with JIA between 2000 and 2015 in New Zealand [6]. The overall incidence rate in their study was comparable to ours (95% CI 4.5–5.7 per 100 000 population in New Zealand, compared with 5.6–6.9 in our UK study). That study found the incidence of JIA was higher amongst CYP of European descent than amongst Maori and Pacific Island children, similar to the white/non-white dichotomous split in our study. Given the wide variety of backgrounds and cultures represented in UK non-white populations, and in New Zealand’s ‘European’ populations, the broad genetics are unlikely to be closely aligned; therefore inequity is more likely than underlying genetic differences as the cause of differential incidence rates between populations. However they found a higher proportion of CYP from low socioeconomic status were diagnosed with JIA in contrast to our own findings. Together, these findings suggest that overall rates of JIA may be similar in New Zealand and the UK (recognizing the difference in study design, with national primary care electronic health records in our study and local hospital records in New Zealand), and differences in rates of JIA diagnosis between ethnic groups may be comparable between nations.

The intersectionality between ethnicity and deprivation, alongside cultural and societal differences between countries, makes interpretation and comparison between the studies challenging given we cannot know all the factors that influence differential incidence rates. Intersectionality also leads to small numbers in subgroups, adding an additional challenge in interpreting results.

A single-centre study in Slovakia [7] reported a higher prevalence of JIA amongst Roma children than the general population, and substantially higher than in our study (112 per 100 000 population overall, compared with 59 per 100 000 in this study). The difference in rates between Roma and non-Roma populations may be due to differences in risk factors, an increased focus on the health needs of Roma communities, or a combination of the two. However, the potential for over-stating the prevalence amongst Roma communities owing to under-representation in the Slovakian census-derived denominators may contribute to this difference.

Finally, a chart-based study in Canada [8] reviewed records of children with inflammatory arthritis between 1976–1980 attending two clinics, and reported a higher prevalence of juvenile arthritis amongst indigenous children than amongst Caucasians, although the primary purpose of that study was to describe two distinct indigenous populations rather than determine prevalence estimates and it is not known if this represents all JIA cases in the community.

Whilst there has been a paucity of studies reporting incidence and/or prevalence of JIA by ethnic group, some studies have given estimates for specific countries. A systematic review of studies across Europe [4] reported a prevalence rate of 70 per 100 000, comparable to the prevalence amongst white CYP in our study. However they also reported wide variation in incidence and prevalence rates based on country, study design and classification.

Whilst a dichotomous ‘white’ and ‘non-white’ analysis is not reflective of ethnic, demographic or social groups, it could potentially highlight the difference in access to care experienced by the two groups. This study has identified that children and young people from non-white ethnic groups are less likely to be diagnosed with JIA, and our analysis also identified that the proportion of children and young people with a specific JIA code by IMD quintiles differed significantly between dichotomous ethnic group, with a much higher proportion of non-white children in the most deprived categories. The proportion of white children diagnosed with JIA is broadly equal by IMD quintiles, whilst it is unevenly distributed amongst non-white children. This may highlight aspects of intersectionality between ethnicity and deprivation in keeping with other data showing higher rates of some autoimmune diseases have been associated with deprivation [20].

The differences we have identified in the incidence and prevalence of JIA between ethnic groups may be due, in part, to biological factors such as different underlying genetics between ethnic groups. However, the genetic heterogeneity between non-white ethnic groups is large, making a genetic white/non-white difference less likely as an explanation for the result shown. Environmental factors (such as pollution or deprivation) are often associated with ethnicity, and could also contribute to the overall disease risk. Finally, there are data demonstrating that different levels of access to healthcare and specialist services exist across ethnic groups. Rapley *et al*. [21] reported that primary care clinicians without experience of JIA are less likely to refer to specialist services; furthermore, Parisi *et al*. [22] found a higher turnover of GPs in more deprived communities (associated with ethnicities), who may have less experience of JIA. This may lead to differential referral rates across ethnicities and therefore apparent differences in incidence rates.

The strengths of this study are that it uses a national dataset with good coverage based in primary care, and is considered broadly representative of the population. The major limitation is that the CPRD does not encode JIA subtypes, which would be a useful addition to consider. Whilst Read codes are not always applied consistently (with a range of codes used to indicate patients with JIA), our robust validation has ensured the number of children with JIA as reported is accurate. In UK practice the vast majority of CYP with JIA will have at least a day case unit admission with an associated ICD-10 code, and/or attend rheumatology or paediatric rheumatology appointments, giving near-complete coverage in the HES-linked data. In addition, JIA is a rare disease so detailed analysis by ethnicity subgroups is not possible due to limited patient numbers.

This study has identified that incidence and prevalence of JIA differs between ethnic groups, and is different from the general population. This may be due to health inequities delaying or preventing a diagnosis of JIA, and is likely to be due to a combination of genetic and equity factors. Further research to understand the underlying cause of these differences is important, to enable targeted interventions and service provision as appropriate.

## Supplementary Material

kead700_Supplementary_Data

## Data Availability

The data in this article were sourced from the CPRD, which is jointly sponsored by the Medicines and Health Regulatory Agency and the National Institute for Health and Care Research (NIHR). The data can be accessed by application to the CPRD at https:///www.cprd.com, where the protocol used to create the dataset can also be found. The Stata code is available upon request from the authors.
