# Supplementary figures and images for: The incidence and prevalence of juvenile idiopathic arthritis differs between ethnic groups in England
Source: Rheumatology (Oxford). 2023 Dec 22;64(1):296–302. doi: 10.1093/rheumatology/kead700 (PMC11701319; doi:10.1093/rheumatology/kead700)

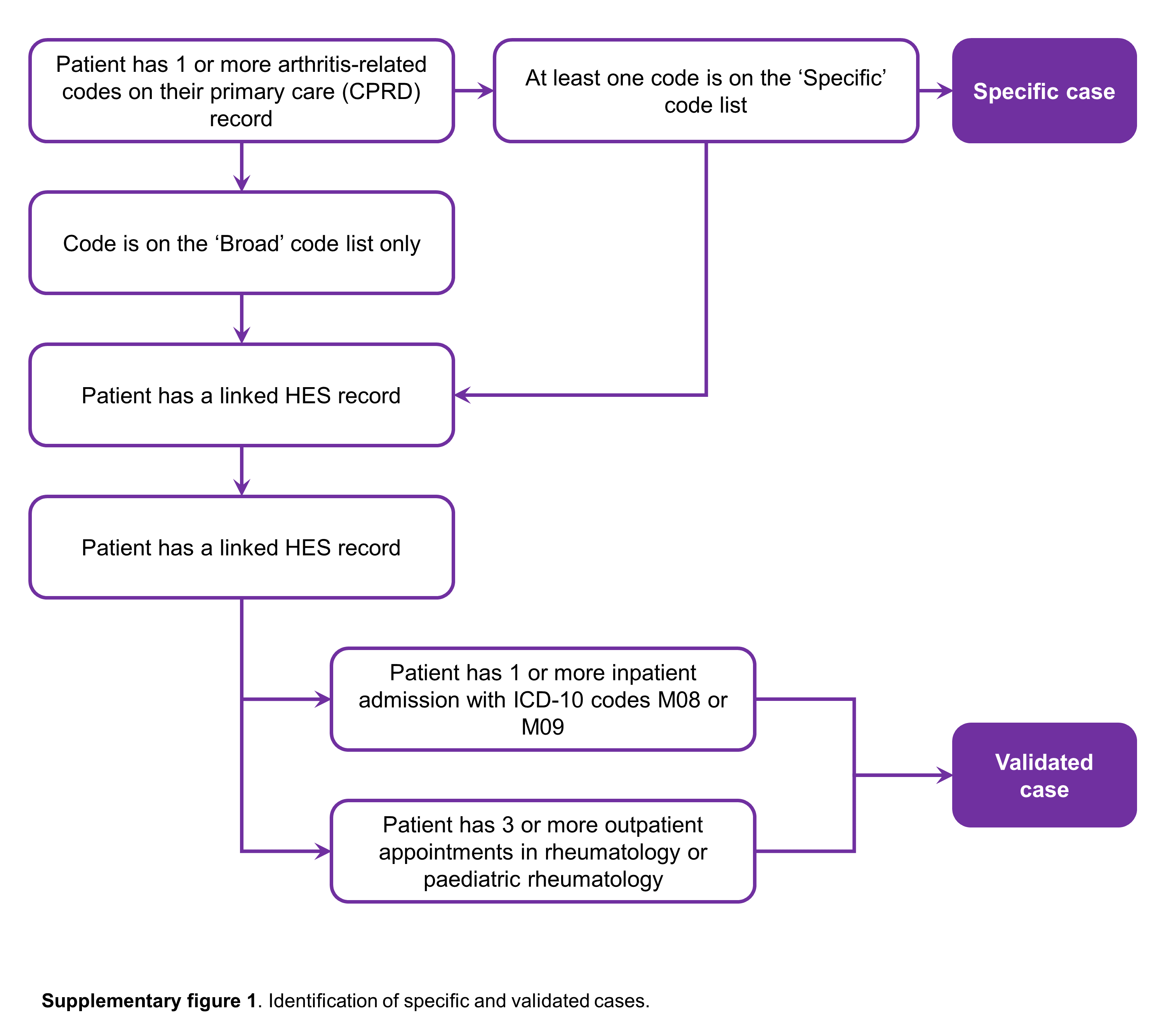

Supplement: kead700_Supplementary_Data [file kead700_supplementary_data.zip › kead700_Supplementary_Data/rhe-23-1106-File006.PNG]

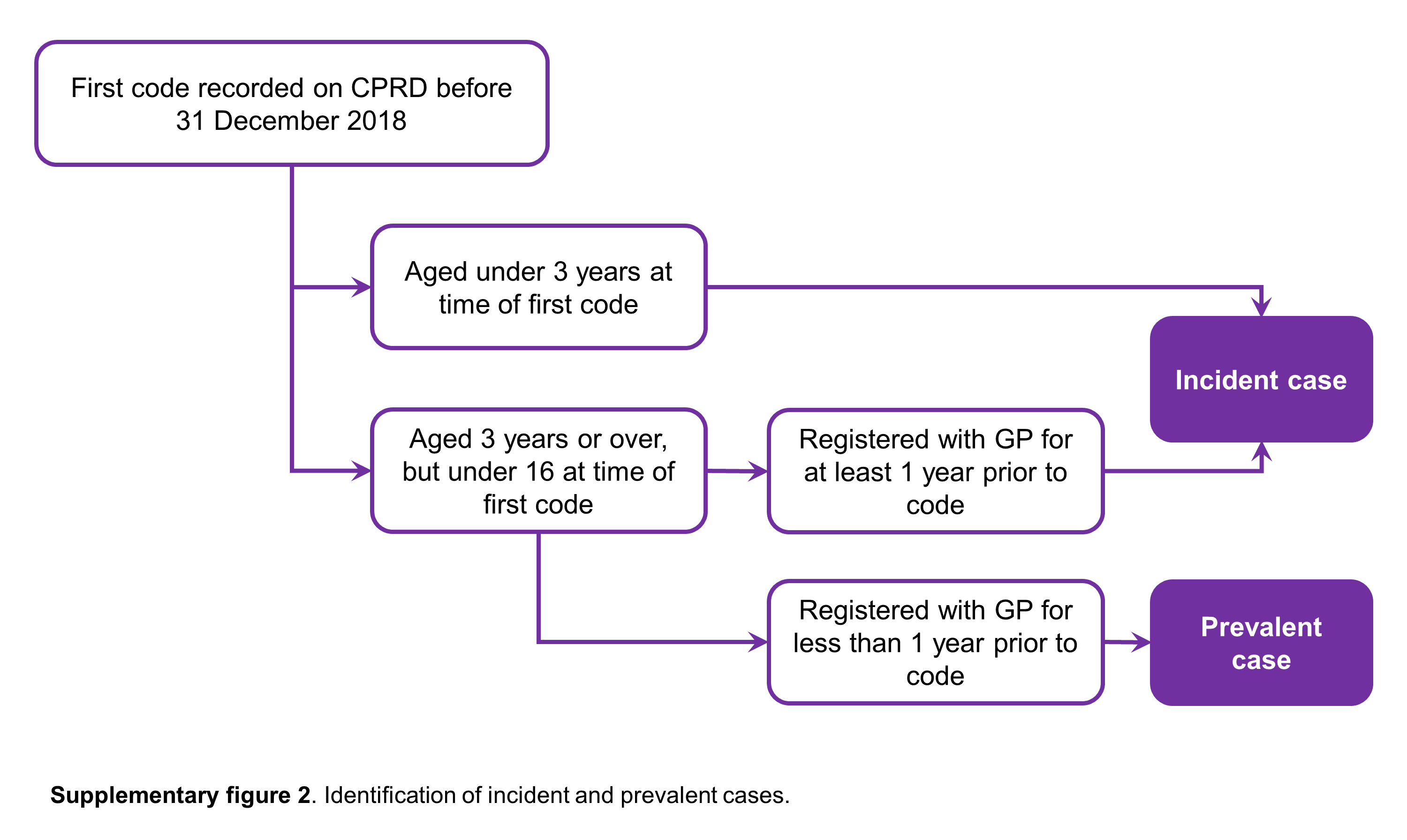

Supplement: kead700_Supplementary_Data [file kead700_supplementary_data.zip › kead700_Supplementary_Data/rhe-23-1106-File007.PNG]
